# l-Type Amino Acid Transporter 1 (LAT1/Lat1)-Utilizing Prodrugs Can Improve the Delivery of Drugs into Neurons, Astrocytes and Microglia

**DOI:** 10.1038/s41598-019-49009-z

**Published:** 2019-09-06

**Authors:** Johanna Huttunen, Soile Peltokangas, Mikko Gynther, Teemu Natunen, Mikko Hiltunen, Seppo Auriola, Marika Ruponen, Kati-Sisko Vellonen, Kristiina M. Huttunen

**Affiliations:** 1 School of Pharmacy, Faculty of Health Sciences, University of Eastern Finland, P.O. Box 1627, FI-70211 Kuopio, Finland; 2 Institute of Biomedicine, Faculty of Health Sciences, University of Eastern Finland, P.O. Box 1627, FI-70211 Kuopio, Finland

**Keywords:** Transport carrier, Transporters, Drug delivery, Transporters in the nervous system, Molecular medicine

## Abstract

l-Type Amino Acid Transporter 1 (LAT1/Lat1) is responsible for carrying large, neutral l-amino acids as well as several drugs and prodrugs across the blood-brain barrier (BBB). However, the BBB is not the only barrier that hinders drugs acting effectively within the brain; the brain parenchymal cell membranes represent a secondary barrier for the drugs with intracellular target sites. In this study, expression and function of Lat1 was quantified in mouse primary neuron, astrocyte and immortalized microglia (BV2) cultures. Moreover, ability of Lat1 to carry prodrugs inside these brain cells was evaluated. The results showed that Lat1 was localized at the similar level in all studied cells (3.07 ± 0.92–3.77 ± 0.91 fmol/µg protein). The transporter was also functional in all three cell types, astrocytes having the highest transport capacity and affinity for the LAT1/Lat1-substrate, [^14^C]-l-leucine, followed by neurons and microglia. The designed prodrugs (**1**-**6**) were able to utilize Lat1 for their cellular uptake and it was mainly much higher than the one of their parent drugs. Interestingly, improved cellular uptake was also achieved in cells representing Alzheimer’s Disease phenotype. Therefore, improved delivery and intra-brain targeting of drugs can be attained by utilizing LAT1/Lat1 and prodrug approach.

## Introduction

Most of central nervous system (CNS) diseases, including neurodegenerative diseases but also neurodevelopmental diseases, lack effective drug therapies. In general, the treatments used against these diseases only diminish symptoms and almost all clinically used drugs have moderate or even severe side effects^1,2^. In addition, the CNS-drug development has slowed down and pharmaceutical companies have withdrawn from this field due to the failures at the late stages of clinical trials^2,3^. One main reason for these failures is lack of efficacy and off-target toxicity of novel CNS-drugs, as the compounds are not been able to cross the structural and functional barrier that protects the brain, namely the blood-brain barrier (BBB)^4^. However, drug brain disposition is highly regulated not only by the BBB, but also by brain cells including neurons and glial cells that serve as a secondary barrier to brain drug exposure^5^. This has not been appreciated in CNS-drug development, although most of the CNS-proteins that could be targeted to produce curative or even preventative medications, are intracellular proteins^6^. Furthermore, there has been lack of understanding of carrier-mediated transport mechanisms as major determinants of drug disposition, action and toxicity^7,8^. Thus, endogenous solute carriers (SLCs) – a family of over 400 proteins – are promising proteins to be utilized to improve drug delivery and targeting to their site of action in the brain.

l-Type amino acid transporter 1 (human ortholog LAT1 and rodent ortholog Lat1) is a transmembrane heterodimeric protein complex, consisting of LAT1 or Lat1 light chain (*SLC7A5 or Slc7a5*) and CD98 heavy chain (4F2hc; *SLC3A2 or Slc3a2*). This protein is selectively and highly expressed at the luminal and abluminal sides of the BBB in relation to other normal tissues^9^. It is a sodium and pH independent transporter and it carries large, neutral, aromatic or branched l-amino acids, such as l-Leu, l-Ile, l-Phe, l-Trp, l-Tyr, l-Met, l-His and l-Val, from systemic circulation across the BBB into the brain. LAT1/Lat1 is an antiporter and as an exchange it transports intracellular amino acids, such as glutamine, with 1:1 stoichiometry across the cell membrane. It has been postulated that the transport rate of LAT1/Lat1 is controlled by the intracellular substrate concentration^10,11^. LAT1/Lat1 is also over-expressed in several types of cancer, supplying cancer cells with their higher demand of amino acids^12–14^. Although LAT1/Lat1 was found to carry not only amino acids, but also drugs such as l-dopa, gabapentin and melphalan in early 2000s^15^, there is still relatively little knowledge of LAT1/Lat1’s role in intra-brain drug disposition. It has been reported that murine primary neurons, astrocytes and microglia express Lat1 mRNA^16^, however, this does not reflect the expression or functionality of the protein on the plasma membrane. This is particularly important to understand, in order to develop intra-brain targeted drugs, i.e., drugs that can in addition of crossing the BBB, also penetrate the secondary barrier of brain parenchymal cell membranes.

Our group has developed LAT1/Lat1-utilizing prodrugs, which can significantly improve the cellular and brain uptake of several parent drugs, such as anti-inflammatory agent ketoprofen^17–19^, anti-epileptic drug valproic acid^20,21^, anti-parkinsonian prodrug of dopamine^22,23^, investigational immunosuppressive perforin inhibitors^24,25^, and natural phenolic antioxidant ferulic acid^26^. We have also recently reported that neither Alzheimer’s disease (AD) induced alterations of transgenic mice nor lipopolysaccharide (LPS)-induced neuroinflammation changed the expression or function of Lat1 at the BBB or primary astrocytes^27,28^. This signifies that LAT1/Lat1 can be utilized for targeted drug delivery not only into the healthy brain but also brain predisposed to pathological changes of brain diseases. However, less is known about LAT1/Lat1 expression or function in other brain cells, like neurons and microglia. Therefore, the aim of the present study was to characterize Lat1 expression and function in mouse primary cortical neurons and immortalized microglia, BV2 cells and compare the results to the ones of primary astrocytes. BV2 cells were chosen for this study as they are accepted alternative for primary microglia since they are more homogenous on target cells than primary populations. Furthermore, it has been reported that the inflammatory response of BV2 cells is, although not identical, very close to the one of primary microglia^29,30^. In this study, we also explored cellular uptake of structurally diverse LAT1/Lat1-utilizing prodrugs, including drugs that can ameliorate neuroinflammation, such as ketoprofen, perforin inhibitors and ferulic acid. Finally, the effects of AD-specific mutations (in genes encoding amyloid precursor protein, *APP* and presenilin, *PSEN1*) on the uptake of these compounds into the primary astrocytes was compared. Thus, the results reported here will increase the current understanding of CNS-drug disposition and highlight the importance of transporters’ role in intra-brain targeted drug delivery that can aid in future CNS-drug development.

## Results and Discussion

### Characterization of brain cells in terms of Lat1 expression and function

**Figure 1:**
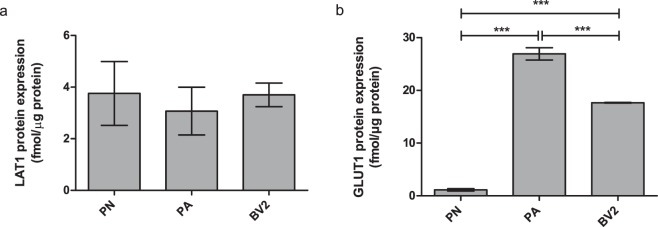
(**a**) Lat1 and **b**) Glut1 expression levels in mouse primary neurons (PN) and astrocytes (PA)^28^, as well as in immortalized microglia (BV2) determined from the crude membrane fractions and measured by SMR/MSM analysis. The data is presented as mean ± SD, n = 3 (***P < 0.001, one-way ANOVA, followed by Tuckey’s multiple comparison test).

Expression levels of Lat1 and glucose transporter 1 (Glut1) in mouse primary neurons and immortalized microglia (BV2) were determined by a selective/multiple reaction monitoring (SRM/MRM) analysis with a liquid chromatography-tandem mass spectrometry (LC-MS/MS) method^31^ and compared to the one of primary astrocytes reported earlier^28^. The expression levels of Lat1 are presented in the Fig. 1a and as shown, all cell types expressed Lat1 within the similar range (3.07 ± 0.92–3.77 ± 0.91 fmol/µg protein). Compared to murine hepatocellular carcinoma cells that are known to overexpress Lat1, the expression level in brain cells was approximately half of that (Lat1 expression in murine hepatocellular carcinoma cells 7.55 ± 0.28 fmol/µg protein)^32^. In comparison, the expression of Glut1 varied between the cell types, being highest in primary astrocytes (26.93 ± 1.16 fmol/µg protein), followed by immortalized microglia (17.65 ± 0.07 fmol/µg protein) and lowest in primary neurons (1.13 ± 0.22 fmol/µg protein) (Fig. 1b), which was in accordance to the literature. It has been reported that only highly glycosylated isoform of Glut1 (55 kDa) is localized in the endothelial cells of the BBB, whereas the less glycosylated isoform of Glut1 (45 kDa) is expressed in glia cells, mainly in astrocytes and oligodendrocytes^33–35^. Neurons, instead, do not express nearly at all any form of Glut1, since glucose is transported into the neurons mainly via Glut3. In addition, neurons can utilize ketone bodies as a back-up energy, provided by the glia cells and transported into the neurons via other carriers, such as monocarboxylic acid transporters (MCTs).

**Figure 2:**
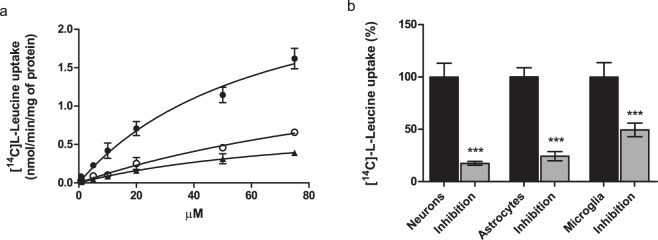
(**a**) Cellular uptake of [^14^C]-L-Leu into the mouse primary neurons (○ open circles) and astrocytes (• filled circles), and immortalized microglias (▴ triangles facing upwards) over a concentration range of 0.76–75 µM. (**b**) Uptake of 0.76 µM [^14^C]-L-Leu into neurons, astrocytes and microglia with and without 100 µM LAT1/Lat1-inhibitor^38^ presented as percentages (%) of [^14^C]-L-Leu uptake, in which the control (100%) is the uptake without the inhibitor. The data is presented as mean ± SD, n = 9 (***P < 0.001, one-way ANOVA, followed by Tuckey’s multiple comparison test).

Function of Lat1 on the cell surface of mouse brain cells was measured with a radiolabeled LAT1/Lat1-probe, [^14^C]-l-leucine, over a concentration range of 0.76–75 µM during 2.5 min incubation time, which was within the linear range of [^14^C]-l-Leu uptake in all studied cell types (evaluated within 0–30 min). Neurons, astrocytes and microglia all transported [^14^C]-l-Leu into the cells with a concentration dependent manner (Fig. 2a). V_max_ and K_m_ values of l-Leu uptake in each cell types are collected in the Table 1, showing that astrocytes transported l-Leu most efficiently across the cell membrane (V_max_ 2.92 ± 0.36 nmol/min/mg of protein and K_m_ value 65.9 ± 14.3 µM). Contrarily, the uptake of l-Leu into primary cortical neurons and immortalized microglia was less effective (V_max_ 0.84–1.90 nmol/min/mg of protein), l-Leu having also lower affinity for Lat1 (K_m_ value 85.8–145.7 µM). Affinity of l-Leu for Lat1 was in all cell types slightly lower than previously reported values (9–52 µM) attained in human LAT1 transfected cells or cancer cells expressing hLAT1 or rodent Lat1^13,36,37^. Relatively high K_m_ values attained in this study may imply that at higher concentrations there is another transport mechanism that start to carry l-Leu into the studied cells in addition to Lat1. However, as expected the transporter’s capacity to carry l-Leu was slightly smaller in the brain cells compared to these overexpressing cells (7–135 pmol/min/mg protein), which in turn indicates that Lat1 is the major carrier of l-Leu into the studied brain cells.

**Table 1:** Michaelis-Menten Kinetic Parameters of [^14^C]-L-Leu Uptake into Mouse Primary Neurons and Astrocytes, and Immortalized Microglia.

Cell Type	V_max_ (nmol/min/mg protein)	K_m_ (µM)	V_max_/K_m_
Primary Cortical Neurons	1.90 ± 0.44	145.7 ± 47.3	0.013
Primary Astrocytes	2.92 ± 0.36	65.9 ± 14.3	0.044
Immortalized microglia	0.84 ± 0.12	85.8 ± 19.4	0.010

The uptake of [^14^C]-l-Leu was also demonstrated to be mediated via Lat1, as the selective LAT1/Lat1-inhibitor (KMH-233)^38^ was able to inhibit its uptake in all cell types (Fig. 2b). As the inhibition was most efficient in neurons and astrocytes (82.53 ± 1.88% and 75.71 ± 4.36%), the primary uptake mechanism of l-Leu into these cells was Lat1. Contrarily, the uptake inhibition was less efficient into the immortalized microglia (50.56 ± 6.48%), which implies that in this cell line l-Leu may also utilize other secondary transport mechanism(s), such as Lat2 (*SLC7A8*)^16,39^ or B^0^AT2 (SBAT1, *SLC6A15*)^40^ when Lat1 is occupied. Together with the protein expression data, these results show for the first time that in addition to astrocytes, neurons and microglia also express functional Lat1 on their cell surface.

### Transporter-mediated uptake of compounds into cells

**Figure 3:**
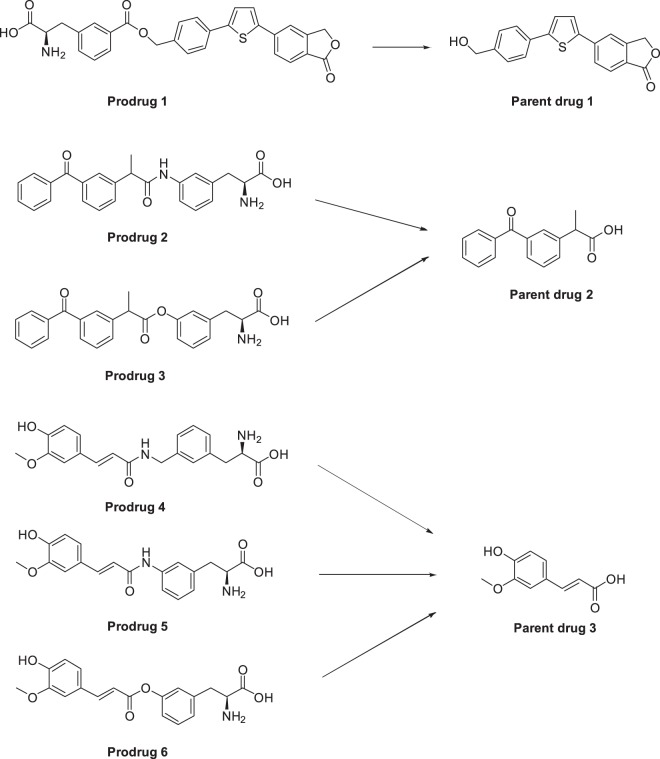
Molecular structures of studied compounds; LAT1/Lat1-utilizing prodrugs **1**–**6** and their maternal drugs **1**–**3**.

**Figure 4:**
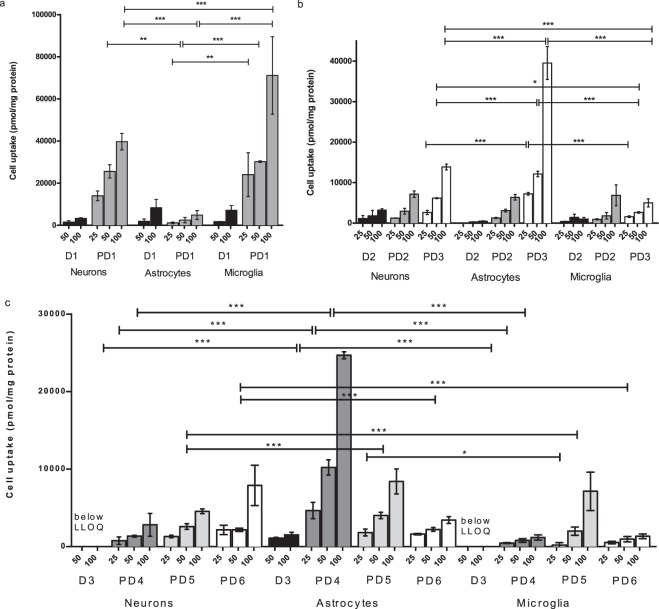
(**a**–**c**) Cellular uptake of 25, 50 and 100 µM prodrugs **1**–**6** (PD1-6) and their parent drugs **1**–**3** (D1-3) into mouse primary neurons and astrocytes, as well as immortalized microglia. The data is presented as mean ± SD, n = 9 and the statistical difference is evaluated only among prodrug uptake into different cell types (*P < 0.05, **P < 0.01, ***P < 0.001, one-way ANOVA, followed by Tuckey’s multiple comparison test).

For the present study we selected 6 house-made LAT1/Lat1-utilizing prodrugs reported earlier. These prodrugs are an investigational immunosuppressive perforin inhibitor (prodrug **1**)^25^, non-steroidal anti-inflammatory drug ketoprofen (prodrugs **2** and **3**)^19^, and natural anti-oxidant ferulic acid (prodrugs **4–6**)^26^ (Fig. 3). They all have been designed and demonstrated to utilize hLAT1 for cellular uptake into the human breast adenocarcinoma cell line (MCF-7) or human retinal pigment endothelial cells (ARPE-19) and to utilize mLat1 for the brain uptake across the mouse BBB^19,24–26^. In this study we wanted to evaluate if these compounds can also utilize Lat1 for their cellular uptake into mouse primary neurons, astrocytes and immortalized microglia. According to the results presented in Fig. 4a–c, all prodrugs were accumulated into the brain cells comparably and most cases even more effectively than their parent drugs in corresponding concentrations (25, 50, 100 µM). The cell viability was also evaluated with microglial cells at the highest exposure concentration (100 µM) for 24 h. Interestingly, prodrugs **4** and **5** slightly increased the cell proliferation, while the other studied compounds did not have any effect on the cell viability (see Supplementary Fig. S2).

Prodrug **1** had 10–17 higher uptake to neurons and microglia than its parent drug **1** (13.98 ± 2.32–71.12 ± 18.42 nmol of PD1/mg protein vs. 1.55 ± 0.64–7.14 ± 2.26 nmol of D1/mg protein). However, the uptake of prodrug **1** into primary astrocytes was overall lower or at the similar range as its parent drug (1.17 ± 0.42–4.88 ± 2.09 nmol of PD1/mg protein vs. 1.93 ± 1.04–8.39 ± 3.85 nmol of D1/mg protein). Thus, prodrug **1** was transported into different cell types in the following order; immortalized microglia > primary cortical neurons > primary astrocytes; the difference being greatest and statistically significant between astrocytes and microglia with all concentrations, and between neurons and astrocytes as well as neurons and microglia only with concentrations higher than 50 and 100 µM, respectively (Fig. 4a). Prodrug **1** has been found to be able to utilize also organic anion transporting polypeptides (OATPs) for its cellular uptake, when LAT1/Lat1 is inhibited or with competitive situation with higher concentrations of this compound^24,25^. Therefore, this result may point out that the ability of utilize secondary transport mechanism may drive this compound to microglia with higher concentrations. Prodrug **1** has also highest molecular weight (Mw. 513.56 g/mol), highest clogP value (2.75) and lowest unspecific brain tissue binding (unbound fraction, f_u_; 1.4%) (Table 2), which may also affect to the accumulation into the microglia. For example, the substrates of Oatps, which are low affinity – high capacity transporters, are relatively lipophilic and big in their size. However, to date there is very little knowledge of localization of functional OATP/Oatp isoforms in neurons and astrocytes, and no published data of OATPs/Oatps in microglia^41^.

**Table 2:** Pharmaceutical Properties of Studied Compounds; LAT1/Lat1-Utilizing Prodrugs **1**–**6** and Their Maternal Drugs **1**–**3**.

Prodrug	Mw (g/mol)	clogP/clogD_7.4_	Brain Protein Binding f_u_ (%)	Maternal Drug	Mw (g/mol)	clogP/clogD	Brain Protein Binding f_u_ (%)
**PD1**	513.56	2.75/3.15	1.4	**D1**	322.38	3.62/3.83	0.03
**PD2**	416.48	0.35/1.88	6.2	**D2**	254.29	2.76/0.46	14.7
**PD3**	417.46	0.99/2.25	n.d.	**D2**	“	“	“
**PD4**	370.41	−0.94/−0.37	13.0	**D3**	194.19	1.42/-1.66	26.0
**PD5**	356.38	−0.85/−0.06	7.0	**D3**	“	“	“
**PD6**	357.36	−0.41/0.54	n.d.	**D3**	“	“	“

Unspecific Brain Tissue Binding Reported as Fraction Unbound (f_u,brain_) has been reported earlier (n.d. = not determined due to the instability of ester prodrugs during the equilibrium dialysis)^19,24^.

Prodrug **1** was detected mainly in its parent drug form as it was chemically hydrolyzed in cell lysate (0.1 M NaOH). However, this compound has been reported to be bioconverted both *in vitro* in brain S9 subcellular fraction, having half-life of approximately 4.7 h, and *in vivo* with half-life ranging from 75 min (liver) to 84 min (brain) and 94 min (plasma)^24^. In immortalized microglia and primary astrocytes the half-lives were 237.51 ± 44.28 min (ca. 4 h) and 348.35 ± 24.13 min (ca. 6 h), respectively, corresponding the one reported earlier in brain S9 subcellular fraction^24^. As the half-life is relatively long, it was concluded that the bioconversion most likely does not affect to selective accumulation of prodrug **1** into the microglia. However, we cannot ignore the possibility of other types of metabolism, which was not followed in this study, as it may also affect to the selective accumulation of prodrug **1** to microglia.

Targeting prodrug **1** into the neurons over other brain cell types would be highly beneficial, since it has been shown that the cytolytic protein, perforin, released by cytotoxic lymphocytes (CTLs) and natural killer (NK) cells, is responsible of mediating the delivery of pro-apoptic serine proteases called granzymes, into the neurons^42^. This mechanism has been found to precede and thus, to induce the neuronal cell death. However, it has also been reported that reactive astrocytes express perforin in postmortem brains of multiple sclerosis, AD and Huntington’s Disease, all representing of ongoing neuroinflammation^43^. Therefore, reactive astrocytes may contribute to the neural attack and loss in addition to CTLs and NKs. However, as the detailed mechanisms of perforin protein in neuropathologies that leads to destruction of neural cells is not well understood^44^, a perforin inhibitor may have a potential role also in microglia.

Prodrug **2** and **3** had similar or little bit higher (1–7-times) uptake into primary cortical neurons and immortalized microglia compared to their parent drug **2**, ketoprofen (Fig. 4b). This was mainly due to the fact that the uptake of ketoprofen was relatively high into the neurons (1.12 ± 0.76–3.20 ± 0.33 nmol of D2/mg protein) but also to lesser extent also to microglia (0.41 ± 0.06–1.45 ± 0.20 nmol of D2/mg protein) and astrocytes (0.14 ± 0.03–0.30 ± 0.01 nmol of D2/mg protein). Ketoprofen is known to utilize primarily organic anion transporters (OAT/Oat) 1, 2 and 3, but also monocarboxylate transporters (MCT/Mct) as low affinity transporters for its cellular uptake^45–47^. Oat1 has been reported to be highly expressed in neurons^48^ but not in microglia or astrocytes, which can explain the higher accumulation of ketoprofen into neurons in this study. However, during neuroinflammation, activated microglia but also astrocytes are the main producers prostaglandin E_2_ via cyclo-oxygenase 2 (COX2)^49^, which ketoprofen is able to inhibit. Thus, the efficacy of ketoprofen must be impaired if it is not transported effectively into its targets cells, microglia and astrocytes, and it raises also questions of off-target toxicity of ketoprofen in neurons.

The uptake into primary astrocytes was 10–80-times higher with both prodrugs **2** and **3** than the one of ketoprofen (1.27 ± 0.14–3.09 ± 0.35 nmol of PD2/mg protein and 7.22 ± 0.32–39.52 ± 4.08 nmol of PD3/mg protein vs. 0.31 ± 0.02–0.49 ± 0.07 nmol of D2/mg protein). However, the uptake of prodrug **2** was almost equivalent to all cell types, while prodrug **3** was accumulated most effectively to primary astrocytes and to primary cortical neurons, having lowest uptake into immortalized microglia. The difference between astrocytes and neurons as well as between astrocytes and microglia was statistically significant already at the concentration of 25 µM (Fig. 4b), implying that the prodrug **3** has higher selectivity for astrocytes over neurons and microglia. However, the uptake of prodrug **3** between neurons and microglia was also statistically significant at 50 µM concentration, indicating the accumulation of prodrug **3** favors neurons over microglia. Curiously, also prodrug **3** is known to be able to utilize other transport mechanism, such as OATPs^50^. However, we have not yet been able to identify if prodrugs **1** and **3** have selectivity for certain subforms of OATPs/Oatps, which could explain their cell-type selectivities that favors microglia and astrocytes, respectively. On the other hand, prodrug **3** being an ester prodrug, is also fully converted to ketoprofen mouse brain S9 subcellular fraction quite rapidly (t_½_ 51.96 ± 2.32 min)^50^. Contrarily, the corresponding amide prodrug **2** is stable and do not release ketoprofen at all, since the *in vitro* media lack co-factors, such as metal ions essential to metalloproteases, potential bioconverting enzymes of amide prodrugs. However, this amide prodrug **2** is able to release ketoprofen *in vivo* situation, as reported earlier (half-life ranging from 16 min (plasma) to 23 min (liver) and 27 min (brain))^19^. The same phenomenon was also detected in microglia and astrocytes, the prodrug **3** having half-lives of 201.25 ± 13.45 min and 282.73 ± 15.80 min, respectively, while prodrug **2** was completely stable in both cell homogenates. Curiously, the half-lives of prodrug **3** in cell homogenates were higher than in brain S9 subcellular fraction. Nevertheless, the bioconversion ability of prodrug **3** may be the reason why it was accumulated into primary astrocytes more effectively than its corresponding amide prodrug **2**. If astrocytes possess higher prodrug hydrolyzing enzyme activity than other brain cell types and since Lat1 facilitates the transport of compounds down to concentration gradient, the uptake of prodrug **3** into the astrocytes may be determined by high bioconversion rate. Another thing that distinguishes the prodrugs **2** and **3** from each other is spatial arrangement; the amide bond of prodrug **3** makes it more rigid than the corresponding ester bond in prodrug **2**, which is more flexible over the entire structure. This may influence the binding and translocation of the compound across the plasma membrane via Lat1.

Taken together, although prodrugs **2** and **3** were accumulated with higher amounts into microglia and astrocytes than ketoprofen itself, they were also be transported into neurons, the non-target cells. Therefore, this prodrug structure is needed to be optimize to achieve higher targeting efficacy into the glia cells over neurons. However, compared to ketoprofen itself, which utilizes Oats for it cellular uptake, changing the transport mechanism to Lat1 would be highly beneficial particularly in the case of neurodegenerative diseases, such as AD, since we have recently reported that Oat3 gene expression is reduced in hippocampus as well as in the microvessels of AD-transgenic mice compared to the corresponding wild type controls, while the gene expression of Lat1 was comparable between the groups^27^. This implies that the delivery of ketoprofen across the BBB and into the target cells may be impaired in AD via Oat3, which could be retained by utilizing LAT1/Lat1- prodrug approach.

The uptake of ferulic acid prodrugs was more complicated, as ferulic acid itself and most likely also the prodrugs **4**–**6** were metabolized to unknown metabolites in primary neurons, astrocytes and immortalized microglia, which we were unable to quantify in this study. Furthermore, the amount of ferulic acid in neurons and microglia remained under the detection limit (1.0 nM). However, the prodrug **4** was more effectively accumulated into the primary astrocytes than primary neurons or immortalized microglia with all studied concentrations (4.65 ± 1.05–24.67 ± 0.45 nmol of PD4/mg protein in astrocytes vs. 0.45 ± 0.06–2.80 ± 1.46 nmol of PD4/mg protein in neurons or microglia) while prodrugs **5** and **6** were more equally distributed over all cell types (0.56 ± 0.19–8.40 ± 1.61 nmol of PD5/mg protein and 0.52 ± 0.15–7.89 ± 2.60 nmol of PD6/mg protein), prodrug **5** favoring glial cells over neurons and prodrug **6** favoring neurons over glial cells at higher concentrations (Fig. 4c). All ferulic acid prodrugs are the smallest of the studied compounds (Mw. 370.41, 356.38 and 357.36 g/mol, respectively), but also have the lowest clogP values (ranging from -0.41 to -0.94). Furthermore, the unspecific brain tissue binding, f_u,brain_ was measured to be highest for prodrug **4** (13%). Prodrug **6** is also an ester prodrug, however, its half-life in brain S9 subcellular fraction is much longer than with other ester prodrugs (**1** and **3**); after 5 h 70% of intact prodrug has been detected^26^, while the half-lives of prodrugs **1** and **3** in brain S9 subcellular fraction were approximately 5 and 1 h, respectively. The slow bioconversion of prodrug **6** was also detected in microglia and astrocytes, estimated half-lives being over 9 h in both cases, while the amide prodrugs **4** and **5** were stable in these *in vitro* conditions, as expected. However, the major determinant for the accumulation of prodrugs **4**–**6** due to the size, lipophilicity, unspecific protein binding or bioconversion cannot be estimated as the unknown metabolites were not evaluated in this study.

Being an antioxidant and a potential β-site amyloid precursor protein cleaving enzymes 1 (BACE1) inhibitor that is mainly functioning in neurons and to lesser extent in astrocytes^51–53^, targeting ferulic acid via its prodrug form into neurons is highly desired property in order to decrease the oxidative stress as well as the β-amyloid formation within the neurons, which both are characteristic features in AD. However, due to the antioxidant properties of ferulic acid, the accumulation into the glial cells would also be beneficial, since most of the oxidative radicals, such as reactive oxygen and nitrogen species (ROS and RNS) are produced in activated glial cells^54–56^.

**Figure 5:**
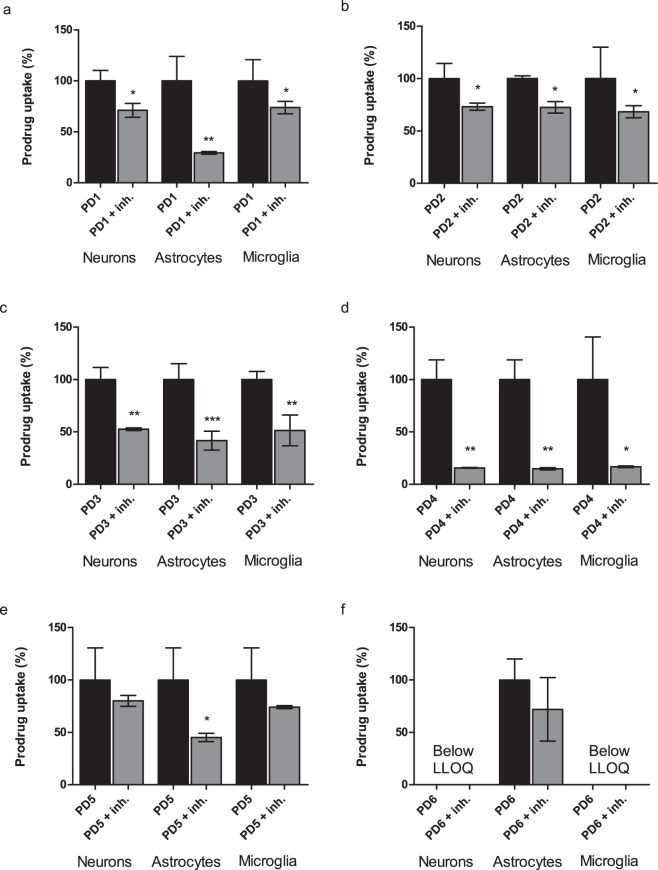
(**a**–**f**) Cellular uptake of 1–10 µM prodrugs **1**–**6** into primary neurons and astrocytes and immortalized microglia in the absence (black bars) and the presence (grey bars) of 100 µM LAT1/Lat1-inhibitor presented as percentages (%) of prodrug uptake, in which the control (100%) is the uptake without the inhibitor. The data is presented as mean ± SD, n = 9 (*P < 0.05, **P < 0.01, one-way ANOVA, followed by Tuckey’s multiple comparison test).

The confirm that the cellular uptake was mediated via Lat1, the uptake of 1–10 µM prodrugs **1**–**3** into primary neurons and astrocytes, and immortalized microglia was studied in the absence and the presence of 100 µM LAT1/Lat1-inhibitor (KMH-233)^38^. 10 µM concentration was used for the prodrugs **4**–**6** due to their higher extent of metabolism in these cells, while other prodrugs were studied with 1 µM concentrations. Despite, the prodrug **6**, which was fully converted to ferulic acid by NaOH in these samples and since the ferulic acid had much higher limit of detection than its prodrugs, we were not able to quantify prodrug **6** in neurons or microglia. However, as seen in the Fig. 5 the cellular uptake of prodrugs **3** and **4** was inhibited significantly by the LAT1/Lat1-inhibitor. The inhibition of prodrug **4** uptake was highest in all cell types (83.24–85.23%) as it was efficiently accumulated into these cells (Fig. 5d). Similar trend was seen with prodrug **3** in all cell types (47.41–58.32% inhibitions; Fig. 5c). All the other prodrugs were less effectively inhibited, which may imply that prodrugs **1**, **2**, **5** and **6** may not be able to compete efficiently with the used novel LAT1/Lat1-inhibitor (Fig. 5a,b,e,f). Overall, it needs to keep in mind that in this study, relatively high concentrations of studied compounds were used, which may also explain lower Lat1-inhibition values. At higher concentrations and when Lat1 is inhibited, there is a possibility that these compounds start to utilize secondary transport mechanisms. Therefore, these results need to be interpreted and translated to the *in vivo* and clinical situations very carefully. Overall, these results are in accordance to our previous studies, in which the LAT1/Lat1-inhibitor has been able to reduce the uptake of the prodrugs in human breast cancer (MCF-7) or retinal pigmented epithelial (ARPE-19) cells by 65–93%^19,26,50^.

**Figure 6:**
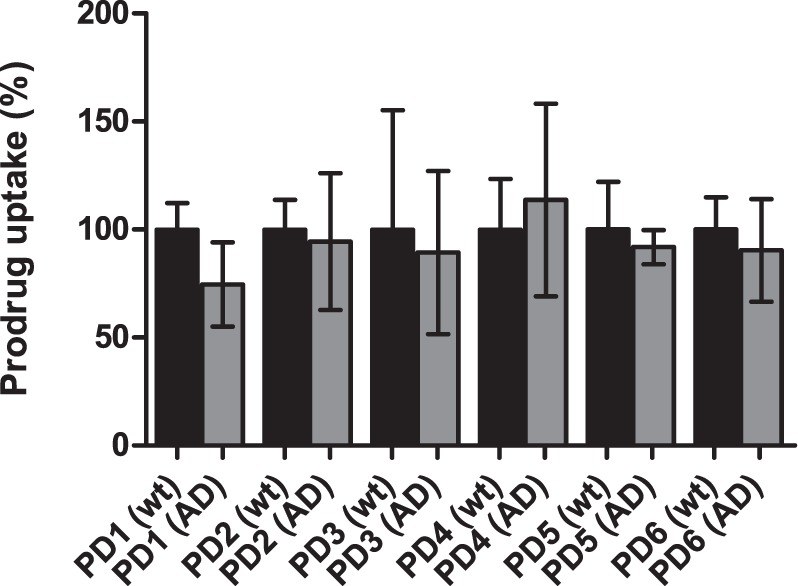
Cellular uptake of 50 µM prodrugs **1**–**6** into primary astrocytes of wild type (wt; black bars) and transgenic AD-astrocytes (grey bars) presented as percentages (%) of prodrug uptake, in which the control (100%) is the uptake without the inhibitor. The data is presented as mean ± SD, n = 9 (no statistically difference, one-way ANOVA, followed by Tuckey’s multiple comparison test).

When comparing the uptake of 50 µM prodrugs **1**–**6** into primary astrocytes isolated from the brain of wild type (wt) and AD transgenic mice, no statistically significant difference was observed between the groups and the uptake into AD-astrocytes were only 3.10–14.18% smaller than into the wt-astrocytes (Fig. 6). This indicates that in addition to the cellular uptake of l-leucine, which we have reported to be retained at the same level in wt-, LPS-induced and AD-astrocytes^28^, the uptake of LAT1/Lat1-utilizing compounds can also be attained regardless of the pathological changes that *APP* and *PS1* gene mutations may cause. This finding is in high importance when developing CNS-prodrugs especially against AD that are attended to act not only as preventative but also as curative medication.

In conclusion, this is the first report to show that primary neurons and immortalized microglia express functional Lat1 protein in addition to primary astrocytes. Furthermore, Lat1 can be utilized to increase the cellular uptake of drugs into the brain parenchymal cells by using a LAT1/Lat1-prodrug approach. Thus, by this approach it is possible to multiply the exposure of target cells to the drugs with therapeutically relevant concentrations and solve one of the main hurdles in CNS-drug development; lack of efficacy has been the greatest single reason (46%) for CNS-drug failure in clinical trials in the past^3^. These results also showed that the increased cellular uptake can be achieved in cells representing AD phenotype with *APP* and *PS1* mutations, as the function of Lat1 was not significantly changed in AD-astrocytes. This is a really important aspect to keep in mind, since some of the transporters, such as GLUT1 or Oat3 can be downregulated in such a conditions^27,28^, which in turn may affect the usefulness of these compounds in clinical situation and thus, impair the CNS-drug development success rate. Moreover, this study shows that by careful prodrug design that takes into account transporter selectivity, brain cell selective drug delivery and targeting between neurons, astrocytes or microglia can be obtained in future, which can lead to improved efficacy and safety of neuroprotective drugs within the brain.

## Experimentals

### Materials

All reagents and solvents used in analytical studies were commercial and high purity of analytical grade or ultra-gradient HPLC-grade purchased from Sigma (St. Louis, MO, USA), J.T. Baker (Denventer, The Netherlands), Merck (Darmstadt, Germany), Riedel-de Haën (Seelze, Germany) or Thermo Fisher Scinetific (Waltham, MA, USA). Water was purified using a Milli-Q Gradient system (Millipore, Milford, MA, USA). LAT1/Lat1-utilizing prodrugs **1**–**6** selected for this study as well as the used LAT1/Lat1-inhibitor (KMH-233) have been published recently^19,25,26,38^, and therefore, their syntheses, identification and purities are not reported herein.

### Cell cultures

Primary embryonic cortical neurons were isolated as previously described^57^. Neonatal (P0‐P2, mixed gender) C57BL/6 WT mice were supplied by the Laboratory Animal Center of the University of Eastern Finland, Kuopio, Finland. The animals were housed in stainless steel cages in controlled environment with 12 h light/dark cycle at an ambient temperature of 22 °C and food and water freely available. The pups were rapidly decapitated, and the brains were dissected and meninges were removed. Brain tissue was dissociated using mechanical shearing and trypsin. The pooled primary cortical neurons were plated (18 × 10^4^/cm^2^) on a poly-D-lysine coated 24-well plates in serum free Neurobasal medium supplemented with 1 × B27, l-glutamine (2 mM) and 1% penicillin (100 U/mL) and streptomycin (100 U/mL). Half of the medium was refreshed after 4 days before performing the experiments described below. Over 80% of this culture has been previously identified to be neurons (12% of astrocytes and 8% of other cell types)^57^.

Primary astrocytes from cortex and hippocampi were isolated from 2-days-old wild type and *APP/PS1* transgenic mice as previously described^58,59^. Mice carrying human *APP* (K595 N and M596 L) and *PSEN1dE9* mutations maintained in C57BL/6 J background were used as a mouse model of AD (Jackson Laboratories, Bar Harbor, ME, USA). The animals were housed and treated as described above, and cortices and hippocampi were isolated by suspending the brain tissue into DMEM medium containing 10% heat-inactivated fetal bovine serum and 100 U/mL penicillin streptomycin. The suspension was triturated ten times and thereafter centrifuged 1500 rpm for 5 min at room temperature. Trypsin-EDTA of 0.25% was added and the suspension was incubated for 30 min at 37 °C. Fresh culture medium was added and the suspension was centrifugated 1500 rpm for 5 min.

The astrocytes were cultured in Dulbecco’s Modified Eagle Medium F-12 Nutrient Mixture (DMEM/F2) supplemented with l-glutamine (2 mM), heat-inactivated fetal bovine serum (10%), penicillin (50 U/mL) and streptomycin (50 µg/mL). The cells were plated on a poly-D-lysine coated flasks in culture medium and to remove the microglia, the cultures were shaken at 200 rpm for 2 h before the experiments described below were performed. It has been reported earlier that these cultures contain approximately 80% astrocytes (20% microglia)^60^. For the cell uptake experiments, the astrocytes (passages 7–16) were seeded on 24-well plates with a density of 10^4^ cells/well three days before the experiments. In the LPS-induced experiments, both wt and AD cells were exposed to 100 ng/ml of LPS for 24 hours before the studied compounds were added on the cells.

Immortalized microglia (BV2) were cultured in RPMI-1640 medium containing l-glutamine (2 mM), heat-inactivated fetal bovine serum (10%), penicillin (50 U/mL) and streptomycin (50 µg/mL). The cells were seeded at the density of 10^5^ cells/well onto 24-well plates one day before the experiments described below by using the passages of 9–13. The function of Lat1 was followed between the used cell passages (astrocytes and microglia) with a LAT1/Lat1 probe substrate, [^14^C]-l-leucine, and noticed to be un-altered.

### Lat1 and Glut1 protein quantitation from brain cells

Crude membrane fractions of mouse primary neurons and astrocytes, as well as immortalized microglia was prepared, and Lat1 and Glut1 protein amounts were quantified by LC-MS/MS by coupling using an Agilent 1290 Infinity LC (Agilent Technologies, Waldbronn, Germany) system to an Agilent 6495 Triple Quadrupole Mass Spectrometer equipped with an ESI source (Agilent Technologies, Palo Alto, CA, USA) as previously described^28^. Data were acquired using the Agilent MassHunter Workstation Acquisition software (Agilent Technologies, Data Acquisition for Triple Quad., version B.03.01) and processed with Skyline software (version 4.2). Example chromatograms for Lat1 and Glut1 from primary neurons and BV2 cells are presented in Supplementary Fig. S1 (see  online). Transporter expression in primary astrocytes was published previously^28^.

### Lat1 functionality and ability of compounds to bind to Lat1

All the uptake studies were done by the same way with each cell type. The cells were carefully washed with pre-warmed HBSS (Hank’s balance salt solution) containing 125 mM choline chloride, 4.8 mM KCl, 1.2 mM MgSO_4_, 1.2 mM KH_2_PO_4_, 1.3 mM CaCl_2_, 5.6 mM glucose, and 25 mM HEPES (pH 7.4 adjusted with 1 M NaOH) after removal of the culture medium. Pre-incubation was done with 500 μL of pre-warmed HBSS at 37 °C for 10 min before adding substrates (250 μL in HBSS) for the uptake experiments. The ability of brain cell to carry a known LAT1/Lat1 substrate, the cells were incubated with [^14^C]-l-leucine at 37 °C for 2.5 min in uptake buffer (HBSS, 250 μL) containing 0.76–75 μM (0.25 mCi/ml) of [^14^C]-l-leucine (PerkinElmer, Waltham, MA, USA). After incubation the reaction was stopped by adding 500 µL of ice-cold HBSS and the cells were washed two times with ice-cold HBSS. The cells were then lysed with 500 μL of 0.1 M NaOH (60 min) and the lysate was mixed with 3.5 mL of Emulsifier safe cocktail (Ultima Gold, PerkinElmer, Waltham, MA, USA). The radioactivity in the cells was measured by liquid scintillation counting (MicroBeta^2^ counter, PerkinElmer Waltham, MA, USA).

The uptake of [^14^C]-l-leucine (0.76 μM) was also studied in the presence of 100 µM LAT1/Lat1-inhibitor (KMH-233) to confirm the Lat1-mediated uptake. The cells were pre-incubated with LAT1/Lat1 inhibitor for 10 min and the incubation mixture was removed before adding [^14^C]-l-leucine and LAT1/Lat1-inhibitor on the cells. The competitive uptake (5 min) and analysis was carried out in a manner similar as described. The inhibition was calculated by comparing the counts in the absence and presence of LAT1/Lat1-inhibitor.

### Transporter-mediated cell uptake of compounds

Cellular uptake of prodrugs **1**–**6** and their parent drugs **1**–**3** (investigational immunosuppressive perforin inhibitor, anti-inflammatory ketoprofen and antioxidant ferulic acid, respectively) were studied by adding compounds at the concentration of 25, 50 or 100 μM in pre-warmed HBSS buffer (250 μL) on the cell layer and incubating at 37 °C for 30 min. Subsequently, the cells were washed three times with ice-cold HBSS and lysed with 500 μl of 0.1 M NaOH (60 min). The supernatants were analyzed by the liquid chromatography mass spectrometry (LC-MS) methods described earlier for prodrug **1** and its parent (perforin inhibitor)^24^, prodrugs **2** and **3** and their parent drug (ketoprofen)^19^, as well as prodrugs **4**–**6** and their parent drug (ferulic acid)^26^, with an Agilent 1200 Series Rapid Resolution LC System together with an Agilent 6410 Triple Quadrupole Mass Spectrometer equipped with an electro-spray ionization source using a Poroshell 120 EC-C-18 column (50 mm × 2.1 mm, 2.7 μm; Agilent Technologies, Santa Clara, CA) for the liquid chromatographic separation of the analytes. The cell-associated concentrations of each compound normalized to protein concentration were calculated from the standard curve that was prepared by spiking known amounts of compounds to ACN including the selected internal standard (diclofenac to all compounds, expect ferulic acid for which chloroxazone was used). The protein concentrations on each plate were determined as a mean of three samples by Bio-Rad Protein Assay, based on the Bradford dye-binding method, using bovine serum albumin (BSA) as a standard protein and measuring the absorbance (595 nm) by multiplate reader (EnVision, Perkin Elmer, Inc., Waltham, MA, USA).

The competitive uptake in the presence of LAT1/Lat1-inhibitor (KMH-233) was carried out as described above with HBSS buffer solution containing 1–10 µM of the studied compounds. The cells were pre-incubated with 100 µM LAT1/Lat1-inhibitor for 10 min and the incubation mixture was removed before adding the studied prodrug and LAT1/Lat1-inhibitor on the cells. The competitive uptake (30 min) with the inhibitor was then carried out as the normal uptake described above. The amounts of studied compounds were analyzed by the LC-MS method and calculated from the spiked standard curve and normalized with the protein concentrations.

### Bioconversion of compounds in immortalized microglia and primary astrocytes

The rates of bioconversion of all studied prodrugs in immortalized BV2 microglia and primary astrocytes homogenates were determined at 37 °C. The cell homogenates were prepared by homogenizing 10 × 10^6^ cells/ml in HBSS (pH 7.4) with SoniPerp 150 Plus disintegrator (MSE Ltd., London, UK) for 2 s × 3. The incubation mixtures were prepared by mixing cell homogenate (final protein concentration 1.0 mg/mL) with HBSS buffer and 10 mM prodrug stock solution in DMSO (the final concentration of prodrugs was 400 µM and the DMSO concentration 2%). The mixture was then incubated for 5 h and the samples (50 µL) were withdrawn at appropriate intervals. The proteins in the samples were precipitated with ice-cold acetonitrile (150 µL) and the samples were centrifuged for 5 min at 12 000 × *g* at room temperature. The supernatants were collected and analyzed by the LC-MS methods as reported earlier^19,24,26^. In blank reactions (chemical stability), the cell homogenate was replaced with same volume of buffer. The pseudo-first-order half-lives (*t*_*½*_) for the rates of bioconversion of the prodrugs were calculated from the slope of the linear portion of the plotted logarithm of remaining prodrug concentration versus time.

### Data analysis

All the studies were carried out as three biological replicates from the different cell passages as well as three technical replicates from the same cell passage. All statistical analyses were performed using GraphPad Prism v. 5.03 software (GraphPad Software, San Diego, CA, USA). Statistical differences between groups were tested using one-way ANOVA, followed by a Tukey’s multiple comparison test and presented as mean ± SD, with statistically significant difference denoted by *P < 0.05, **P < 0.01, ***P < 0.001. clogP values (Table 2) were calculated by using ChemDraw Professional software (PerkinElmer Informatics, Inc., Waltham, MA, USA), and clogD_7.4_ values by using ChemAxon logD predictor (ChemAxon, Ltd. Budapest, Hungary).

### Ethical statement

The experimental procedures involving animals (primary neuron and astrocyte isolation) were made in compliance with the European Commission Directives 2010/63/EU and 86/609, and approved by the Institutional Animal Care and Use Committee of University of Eastern Finland (Animal Usage Plan numbers: EKS‐008‐2016, EKS‐006‐2017 and ESAVI/3347/04.10.07/2015). All efforts were made to minimize the number of animals used and to minimize their suffering.

## Supplementary information

Supplementary Info

## Data Availability

All data generated or analyzed during this study are included in this published article.
