# Supplementary material for: l-Type Amino Acid Transporter 1 (LAT1/Lat1)-Utilizing Prodrugs Can Improve the Delivery of Drugs into Neurons, Astrocytes and Microglia
Source: Sci Rep. 2019 Sep 6;9:12860. doi: 10.1038/s41598-019-49009-z (PMC6731241; doi:10.1038/s41598-019-49009-z)
Supplement: Supplementary file 1 — Supplementary Info [file 41598_2019_49009_MOESM1_ESM.pdf]

Supplementary Information

L-Type Amino Acid Transporter 1 (LAT1/Lat1)-Utilizing Prodrugs Can Improve the Delivery of Drugs into Neurons, Astrocytes and Microglia

Johanna Huttunen,[1] Soile Peltokangas,[1] Mikko Gynther,[1] Teemu Natunen,[2] Mikko Hiltunen,[2] Seppo Auriola,[1] Marika Ruponen,[1] Kati-Sisko Vellonen,[1] Kristiina M. Huttunen [1,]*

[1] School of Pharmacy, Faculty of Health Sciences, University of Eastern Finland,

P.O. Box 1627, FI-70211 Kuopio, Finland

[2] Institute of Biomedicine, Faculty of Health Sciences, University of Eastern Finland,

P.O. Box 1627, FI-70211 Kuopio, Finland

* Corresponding author, E-mail: kristiina.huttunen@uef.fi

Contents

1. Lat1 and Glut1 Protein Quantitation from Brain Cells S2

2. Effects of LAT1-Utilizing Prodrugs and Their Parent Drugs on the Cell Viability of Immortalized Microglia (BV2) S3

1. *Lat1 and Glut1 Protein Quantitation from Brain Cells*

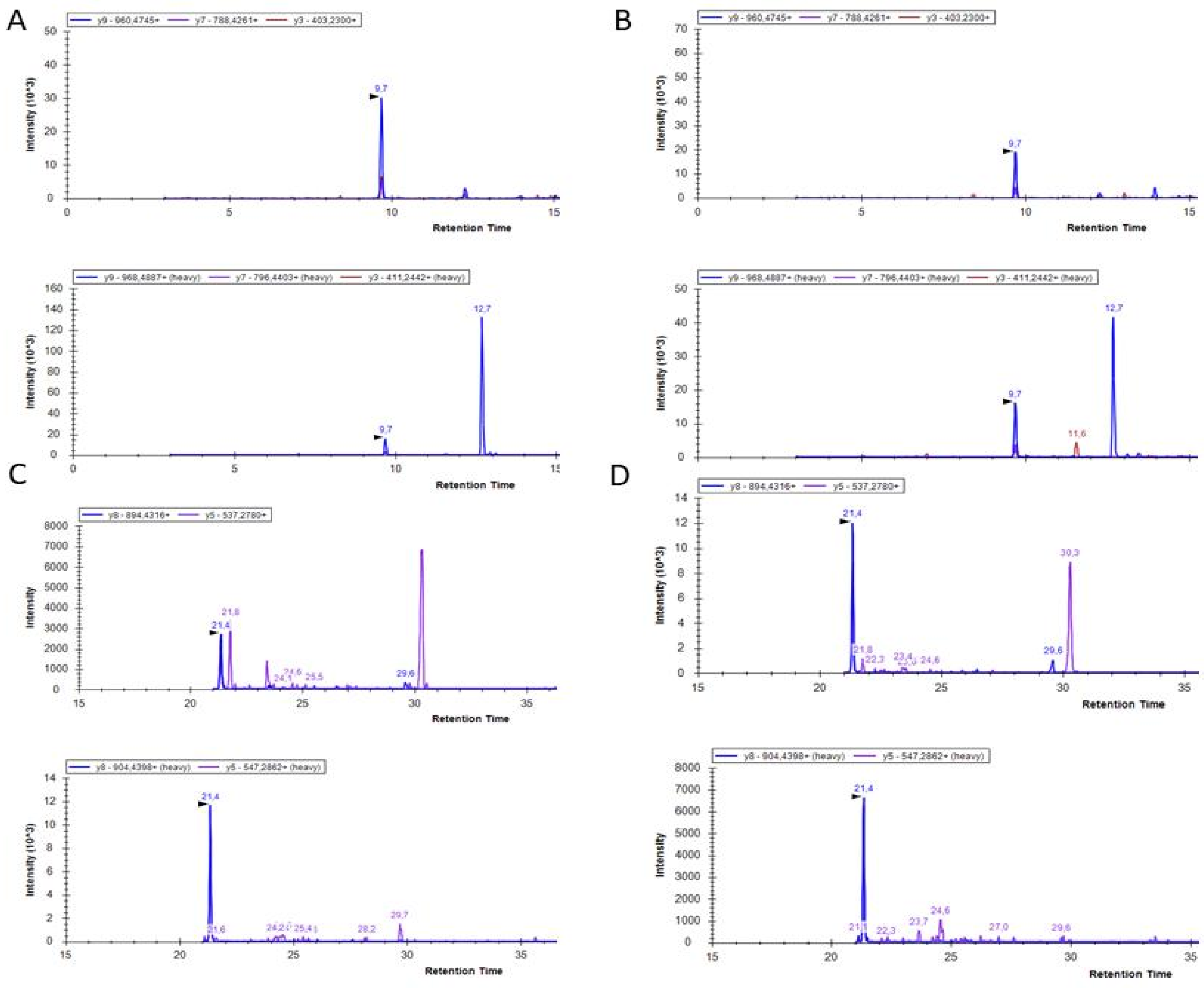

Figure S1. Examples LC-MS/MS chromatograms for protein quantitation generated by Skyline software (version 4.2). Lat1 natural (top panel) and isotopically labeled standard (lower panel) peptide chromatograms in primary neurons (A) and in BV2 cells (B). Glut1 natural (top panel) and isotopically labeled standard (lower panel) peptide chromatograms in primary neurons (C) and in BV2 cells (D).

2. Effects of LAT1-Utilizing Prodrugs and Their Parent Drugs on the Cell Viability of Immortalized Microglia (BV2)

Immortalized microglia (BV2) were seeded at the density of 2 × 10^4 cells/well onto collagen-coated 96-well plates. The cells were used for the proliferation experiments one day after seeding. A concentration of 100 µM of studied compounds were added into the growth medium and incubated for 24 h, after which the cell viability was determined by resazurin cell proliferation kit (Sigma, St. Louis, MO, USA). The samples were measured fluorometrically by monitoring the increase in fluorescence at a wavelength of 590 nm using an excitation wavelength of 560 nm (EnVision, Perkin Elmer, Inc., Waltham, MA, USA), which is directly proportional to aerobic respiration and cellular metabolism of cells. The cell death was confirmed in the decrease of cell amount by visualizing the wells with microscopy. None of the studied compounds decreased the cell viability (**Figure S2**).

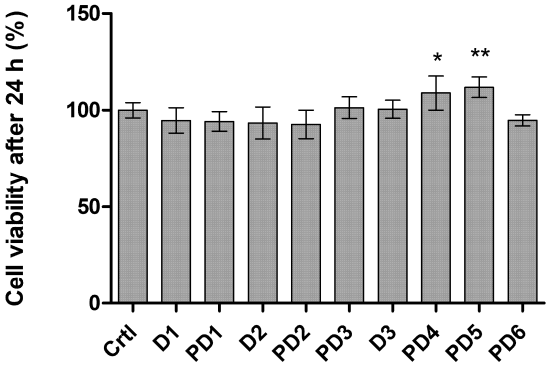

Figure S2. The cell viability of immortalized microglia (BV2) after 24 h incubation of 100 µM LAT1-utilizing prodrugs (PD1-PD6) and their parent drugs (D1-D3) presented as percentages (%) compared to the untreated cells (ctrl) (mean ± SD, n=3-6). An asterisk denotes a statistically significant difference from the respective control (** $P < 0.01$, * $P < 0.05$ one-way ANOVA, followed by Tukey's test).
